# Placental Malaria: Decreased Transfer of Maternal Antibodies Directed to *Plasmodium falciparum* and Impact on the Incidence of Febrile Infections in Infants

**DOI:** 10.1371/journal.pone.0145464

**Published:** 2015-12-23

**Authors:** Celia Dechavanne, Gilles Cottrell, André Garcia, Florence Migot-Nabias

**Affiliations:** 1 Institut de Recherche pour le Développement (IRD), UMR 216 Mère et enfant face aux infections tropicales, Université Paris Descartes, Paris, France; 2 COMUE Sorbonne Paris Cité, Université Paris Descartes, Faculté des Sciences Pharmaceutiques et Biologiques, Paris, France; Johns Hopkins Bloomberg School of Public Health, UNITED STATES

## Abstract

The efficacy of mother-to-child placental transfer of antibodies specific to malaria blood stage antigens was investigated in the context of placental malaria infection, taking into account IgG specificity and maternal hypergammaglobulinemia. The impact of the resulting maternal antibody transfer on infections in infants up to the age of 6 months was also explored. This study showed that i) placental malaria was associated with a reduced placental transfer of total and specific IgG, ii) antibody placental transfer varied according to IgG specificity and iii) cord blood malaria IgG levels were similar in infants born to mothers with or without placental malaria. The number of malaria infections was negatively associated with maternal age, whereas it was not associated with the transfer of any malaria-specific IgG from the mother to the fetus. These results suggest that i) malaria-specific IgG may serve as a marker of maternal exposure but not as a useful marker of infant protection from malaria and ii) increasing maternal age contributes to diminishing febrile infections diagnosed in infants, perhaps by means of the transmission of an effective antibody response.

## Introduction

During pregnancy, the sequestration of *Plasmodium falciparum*-infected erythrocytes in the placenta is known to decrease maternofetal antibody exchange. Several studies have demonstrated an association between placental malaria (PM) and decreased maternal antibody transfer to the fetus, taking into account maternal antibody levels at delivery [1–3]. One of these studies, enrolling 213 mother/child pairs in The Gambia, showed that prematurity and low birth weight were associated with a PM-dependent decrease of *Haemophilus influenzae-* and *Streptococcus pneumoniae*-specific antibody transfer [2]. The authors speculated that this decrease in maternal antibody transfer weakened the infants, making them more vulnerable to bacterial infections [2]. The authors of the above-cited studies showed that the efficacy of the maternal antibody transfer in patients with PM varied according to antibody specificity [1–3]. Okoko *et al*. demonstrated that PM was associated with decreased anti-measles but not anti-tetanus toxoid antibody transfer in the same cohort [2]. In contrast, Scott *et al*. showed that PM was not associated with a lower anti-measles antibody level [4]. Deloron *et al*. observed no difference in the transfer of antibodies targeting *P*. *falciparum* extracts according to placental infection [5]. The effect of PM in specific antimalarial antibody transfer has not been completely clarified.

The transport of IgG across the placenta is an active and selective process specifically mediated by the neonatal Fc receptor (FcRn). One study revealed that high total and anti-measles antibody levels in maternal peripheral blood were associated with reduced efficacy of specific antibody transfer [6]. Saturation of the limited number of FcRn receptors on syncytiotrophoblasts could be generated by high maternal IgG levels [7–9] and may therefore introduce a bias in the analysis of PM’s effect on the efficacy of maternal-specific antibody transfer. Maternal hypergammaglobulinemia is thus an essential factor to consider [1–3], and it was subsequently determined and introduced in the analyses of the present study.

Few studies have investigated the role of transferred maternal antibodies on infants’ clinical protection from malaria. Wilson *et al*. showed that functional anti-malaria antibodies crossed the placenta, by assessing their functionality using the parasite growth inhibition assay [10]. However, another study showed a positive association between maternal antibody transfer and malaria incidence in the 1^st^ year of life [11]. Obviously, the mechanisms determining maternal antibody transfer and its impact on infants’ malaria infections remain unclear.

In the present study, we investigated serum reactivity against immunogenic antigens from *P*. *falciparum* blood stages representing promising vaccine candidates—Apical Membrane Antigen 1 (AMA1), Merozoite Surface Protein (MSP) 1–19, MSP2 (two allelic families: 3D7 and FC27), MSP3 and Glutamate-Rich Protein (GLURP, two regions: R0 and R2)–to highlight the effect of PM in specific antimalarial antibody transfer, considering IgG specificity and maternal hypergammaglobulinemia. The present study also investigated whether lower maternal antibody transfer has consequences on infant health.

## Material and Methods

### Study design and sample collection

The study took place from June 2007 to January 2010 in Southern Benin (Tori Bossito district). A weekly follow-up of 535 infants from birth to 18 months is detailed in a previous publication [12]. The present work focused on the first 6-month period. Maternal circulating blood (CIRC) and cord blood (CORD) samples were collected in Vacutainer^®^ EDTA (Ethylene diaminetetraacetic acid) tubes at delivery and placental blood smears (from the maternal side of the placenta) were made. Plasma was stored at −80°C. Infections were defined as any febrile event (≥37.5°C) and malaria infection was defined as a febrile event with a positive rapid diagnostic test or a positive thick blood smear. Symptomatic malaria infections were treated with artemether/lumefantrine combination therapy, as per the National Malaria Control Program (Benin) recommendations.

### Data collection

The following data were collected: gestational age (estimated using the Ballard method [13], gravidity (primigravid/multigravid), maternal age, maternal weight before delivery, low infant birth weight (defined as a birth weight ≤2500 g, [14]) and presence/absence of PM (defined by the presence of asexual forms of *P*. *falciparum* in thick placental smears). Table 1 shows a summary of these variables, according to presence/absence of PM.

**Table 1:** Characteristics of the population group.

Variables		n (%)	No placental malaria (*n* = 481)	With placental malaria (*n* = 54)	*p*-value
Hypergammaglobulinemia^a^	< 16 g.L^-1^	366 (68.4)	329 (89.9)	37 (10.1)	0.986
	≥ 16 g.L^-1^	169 (31.6)	152 (89.9)	17 (10.1)	
Gravidity^a^	primigravid	82 (15.3)	67 (81.7)	15 (18.3)	**0.007**
	multigravid	453 (84.7)	414 (91.4)	39 (8.6)	
Mother's weight (kilograms)^b^		535 (100)	61.8 [47.5;76.1]	57.9 [51.3;64.5]	0.053
Mother's age (years)^b^		535 (100)	27.7 [22.2;33.2]	25.6 [19.5;31.6]	**0.007**
Low birth weight (grams)^a^	< 2500	48 (9.0)	40 (83.3)	8 (16.7)	0.113
	≥ 2500	487 (91.0)	441 (90.6)	46 (9.4)	
Gestational age (weeks)^b^		535 (100)	38.5 [36.7;40.3]	38.3 [36.5;40.1]	0.437

^a^: Chi_2_ test;

^b^: Student unpaired *t*-test;

percentages are written in parentheses; standard deviations are written in square brackets; in bold: *p*<0.05.

### P. falciparum recombinant antigens

MSP1-19 (Uganda-Palo-Alto strain) and MSP3 (F32 strain) were produced at the Pasteur Institute (Paris, France). MSP2 (3D7 and FC27) were donated by collaborators from La Trobe University (Melbourne, Australia). GLURP-R0 (F32 strain) and GLURP-R2 (F32 strain) were produced by the Infection-Immunity Department of the Statens Serum Institute of Copenhagen (Denmark) and AMA1 was donated by the Biomedical Primate Research Centre (Rijswijk, The Netherlands). All recombinant proteins were expressed in *Escherichia coli* except AMA1, in *Pichia pastoris*.

### Measurement of antibodies against P. falciparum recombinant antigens

Enzyme-Linked ImmunoSorbent Assay (ELISA) [15,16] was performed to assess malaria antibody concentrations; Afro Immuno Assay (AIA) protocols were followed that are developed to standardize methods for evaluating malaria vaccines and sponsored by the African Malaria Network Trust (AMANET [www.amanet-trust.org]). Briefly, standard curves were established using purified human IgG (Binding Site, France) to determine the concentration of specific antibodies. Each point was tested in duplicate.

Specific and total IgG were measured using recombinant proteins diluted in phosphate-buffered saline (PBS) for specific IgG or an anti-human IgG (Fab-specific, Sigma Aldrich, France) diluted in carbonate buffer for total IgG, both were coated at 0.1-μg/well on MaxiSorp Nunc plates (Thermo Fisher Scientific, Denmark) and blocked with 3% powdered milk, 0.1% Tween, 20 PBS. Maternal and cord blood samples were diluted at 1:200 for all IgG-specific, recombinant proteins except for AMA1 (1:2,000) and total IgG (1:1,000,000). An anti-human IgG coupled with peroxidase (1:3,000, Caltag, UK) was used to reveal the reaction with TMB One (3, 3′, 5, 5′-tetramethylbenzidine, Mast Diagnostic, France). Plates were read at 450 nm.

### Management of ELISA data

ELISA optical densities (OD) were analyzed with ADAMSEL FLP b039 software (http://www.emvda.org/portfolio/project-index/optimalvac-project-completed), to determine antibody concentrations (μg/mL). Discordant duplicates (with a variation coefficient >15%) were re-tested.

A stochastic expectation maximization algorithm [17], already applied to ELISA analyses [18], was used to impute data in cases where the OD was below detection thresholds or was oversaturated [referred to as “Low” and “High” concentration values (μg/mL), respectively].

### Placental antibody transfer

Prior to analyses, antibody levels were log-transformed (natural logarithm (ln)). Placental antibody transfer was defined as the natural logarithm of the ratio between neonatal and maternal antibody levels expressed in μg/mL (ln (CORD/CIRC)).

### Statistical analyses

For quantitative variables, means were compared using a Student unpaired *t*-test. A chi-squared test was used to compare categorical variables (Table 1). Linear regression was used for both univariate and multivariate analyses of the antibody transfer. Analyses were adjusted according to maternal-specific IgG levels, hypergammaglobulinemia (defined as total IgG ≥16 g.L^−1^ [1–3]) and the variables listed in the “Data collection” section. A logistic regression was used for both univariate and multivariate analyses of the impact of the maternal antibody transfer on infant infections. A multinomial logistic model investigated the effect of maternal antibody transfer on malaria or non-malaria infections in the infants. Multivariate analysis was performed, including the variables with *p*≤0.20 in the univariate step. Statistical significance was set at *p*<0.05 in the linear regression analysis and at *p*<0.01 in the logistic regression. All statistical analyses were performed using Stata, version 13.0 (StatCorp LP, College Station, TX, USA).

### Ethics

This study protocol was approved by the institutional Ethics Committee of the Faculté des Sciences de la Santé from the Université d’Abomey-Calavi in Benin and the Comité Consultatif de Déontologie et d’Ethique from the French Institut de Recherche pour le Développement. The nature of the project was explained in detail to the participants and informed consent was obtained from the women included in the study. All women signed informed consent before enrollment (which also included their children) and could withdraw from the study at any time [12].

## Results

### Description of the cohort

Ten percent of the pregnant women enrolled had an active placental infection at delivery (*n* = 54). Maternal hypergammaglobulinemia, maternal weight, infant gestational age and low infant birth weight were not affected by PM. The risk of PM was higher in primigravid mothers and in younger mothers (Table 1).

### IgG levels in maternal peripheral blood and cord blood

Maternal-specific IgG levels were higher at delivery in women presenting with PM than those without PM. This difference was significant for AMA1, MSP1-19, MSP2-3D7 and MSP3 (all *p*<0.049; Table 2). Interestingly, most antimalarial IgG levels in cord blood were similar, whether or not the mother presented with PM at delivery, with the exception of higher IgG levels to MSP2-FC27 (*p* = 0.035) and lower total IgG levels (*p* = 0.026) in PM infection (Table 2).

**Table 2:** Cord blood and maternal specific antibody levels as well as their ratio, according to placental malaria infection.

Antigen	Bloodcompartment	IgG levels ^b^		*p*-value
		No placental infection (*n* = 481)	With placental infection (*n* = 54)	
AMA1	CIRC ^a^	577.81 [573.68;581.93]	897.33 [893.61;901.04]	**0.03**
	CORD ^a^	518.62 [514.95;522.28]	667.16 [663.44;670.87]	0.178
	(CORD/CIRC) ^a^	0.89 [-0.69;2.46]	0.74 [-0.87;2.36]	**0.007**
MSP1-19	CIRC	29.91 [22.65;37.17]	51.50 [46.41;56.58]	0.053
	CORD	22.53 [16.55;28.50]	27.37 [22.83;31.90]	0.442
	(CORD/CIRC)	0.76 [-1.03;2.55]	0.53 [-1.35;2.41]	**<0.001**
MSP2-3D7	CIRC	84.64 [80.32;88.96]	130.34 [126.52;134.16]	**0.039**
	CORD	47.16 [43.35;50.97]	58.23 [54.91;61.56]	0.268
	(CORD/CIRC)	1.79 [-0.12;3.71]	2.24 [0.34;4.14]	**0.018**
MSP2-FC27	CIRC	50.09 [46.81;53.37]	65.79 [62.90;68.69]	0.107
	CORD	94.01 [89.70;98.32]	145.62 [142.07;149.17]	**0.035**
	(CORD/CIRC)	1.88 [-0.08;3.83]	2.21 [0.32;4.11]	0.085
MSP3	CIRC	8.17 [2.50;13.83]	13.99 [8.41;19.59]	**0.031**
	CORD	5.50 [0.42;10.59]	7.33 [2.50;12.15]	0.22
	(CORD/CIRC)	0.67 [-1.01;2.35]	0.52 [-1.11;2.15]	**0.001**
GLURP-R0	CIRC	5.49 [0.63;10.36]	7.41 [2.31;12.51]	0.189
	CORD	3.36 [-1.07;7.80]	3.46 [-1.17;8.09]	0.894
	(CORD/CIRC)	0.61 [-1.11;2.33]	0.47 [-1.13;2.06]	**0.001**
GLURP-R2	CIRC	36.97 [33.04;40.90]	54.62 [49.45;59.78]	0.052
	CORD	21.47 [17.89;25.06]	22.70 [18.73;26.68]	0.763
	(CORD/CIRC)	0.57 [-1.15;2.29]	0.42 [-1.67;2.51]	**<0.001**
Nonspecific total IgG	CIRC	12096.24 [12093.78;12098.71]	10943.12 [10940.42;10945.81]	0.444
	CORD	10703.50 [10701.21;10705.78]	8177.39 [8174.82;8179.97]	**0.026**
	(CORD/CIRC)	0.88 [-1.21;2.98]	0.75 [-1.13;2.63]	0.105

^a^: CIRC: maternal peripheral blood at delivery; CORD: cord blood, (CORD/CIRC): ratio representing the transfer of maternal IgG to the neonate at birth;

^b^: geometric mean [95% confidence interval] of the IgG concentrations;

Student’s unpaired *t*-test was performed; n: effective; in bold: *p*< 0.05.

### Placental transfer of malaria-specific IgG

**Fig 1:**
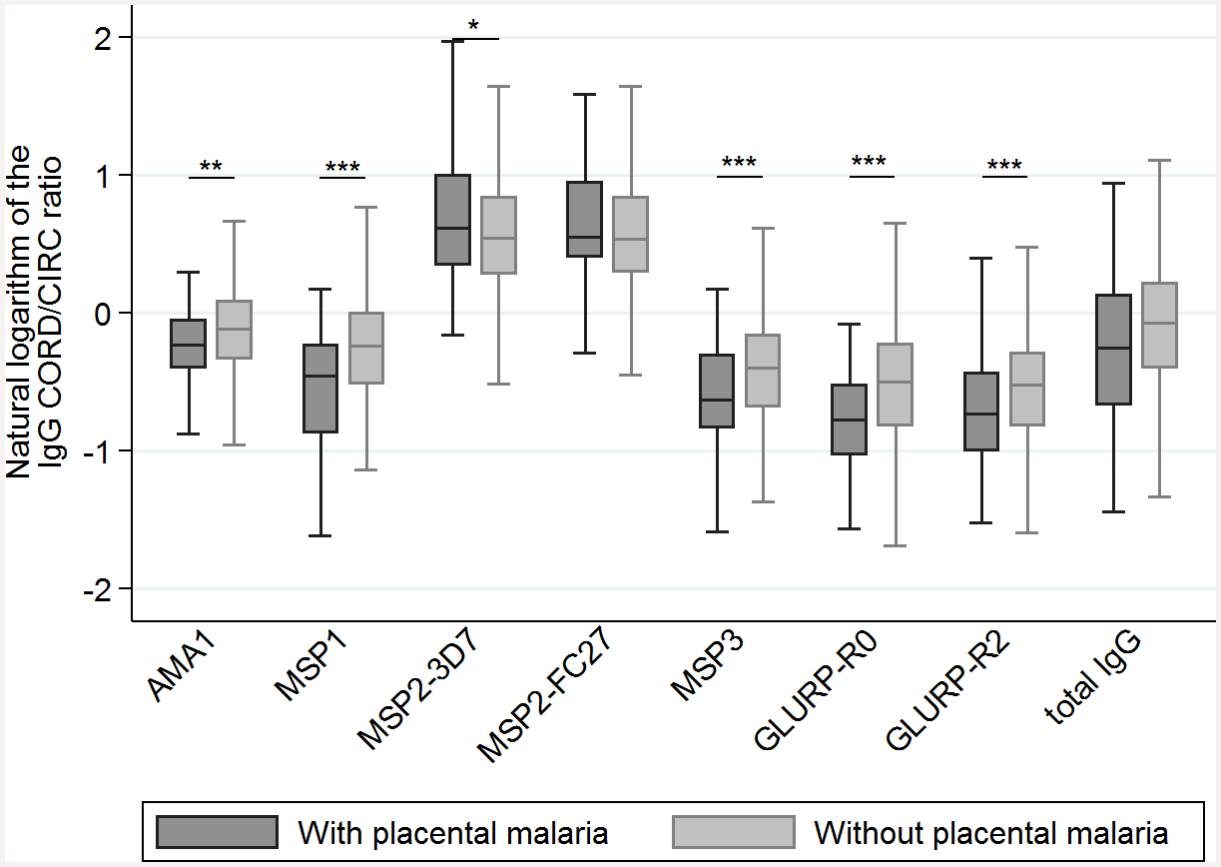
Cord-to-mother IgG transfer according to placental malaria. CIRC: maternal peripheral blood at delivery; CORD: cord blood, ln(CORD/CIRC): ratio representing the transfer of maternal IgG to the neonate at birth; * <0.05; ** ≤0.01; *** ≤0.001.

Placental transfer of malaria-specific IgG was significantly reduced in the presence of PM (all *p*<0.019) for all antigens except MSP2-FC27 and total IgG (Table 2, Fig 1). PM and maternal-specific IgG levels were the two main factors consistently associated with the reduction of placental transfer, except MSP2 (PM) and total IgG (maternal IgG levels) (Table 3). Maternal hypergammaglobulinemia impaired the transfer of some specific IgG (directed to AMA1, MSP2-FC27 and GLURP-R2) and total IgG from the mother to the fetus (Table 3). Other factors were occasionally associated with the CORD/CIRC ratio: the transfer of IgG specific to MSP2-3D7 was positively associated with gestational age, maternal age and maternal-specific IgG levels. The transfer of MSP1 was negatively associated with gestational age.

**Table 3:** Linear regression model showing the factors involved in impaired maternal IgG transfer.

	AMA1		MSP1		MSP2-3D7	
Variables	coeff^a^	*p*-value	coeff	*p*-value	coeff	*p*-value
Placental malaria	-0.126 [-0.245;-0.006]	0.039	-0.293 [-0.44;-0.145]	<0.001		
Gestational age			-0.029 [-0.054;-0.004]	0.023	0.265 [0.096;0.433]	0.002
Maternal age					0.016 [0.005;0.027]	0.004
Maternal-specific IgG	-0.118 [-0.144;-0.092]	<0.001	-0.135 [-0.158;-0.112]	<0.001	0.187 [0.152;0.221]	<0.001
Hypergammaglobulinemia	-0.097 [-0.176;-0.018]	0.016				
	MSP2-FC27		MSP3		GLURP-R0	
Variables	coeff	*p-value*	coeff	*p-value*	coeff	*p-value*
Placental malaria			-0.188 [-0.324;-0.051]	0.007	-0.232 [-0.375;-0.089]	0.002
Gestational age						
Maternal age						
Maternal-specific IgG	0.084 [0.037;0.132]	<0.001	-0.107 [-0.13;-0.083]	<0.001	-0.113 [-0.141;-0.086]	<0.001
Hypergammaglobulinemia	0.2 [0.08;0.319]	0.001				
	GLURP-R2		Total IgG			
Variables	coeff	*p-value*	coeff	*p-value*		
Placental malaria	-0.252 [-0.398;-0.106]	0.001				
Gestational age						
Maternal age						
Maternal-specific IgG	-0.153 [-0.185;-0.121]	<0.001				
Hypergammaglobulinemia	-0.157 [-0.253;-0.061]	0.001	-0.581 [-0.705;-0.458]	<0.001		

^a^: coeff [95% confidence interval]: coefficient determined by the linear model. A positive (negative) coefficient corresponds to a positive (negative) association between two variables.

### Maternal antibody transfer and infant infections

Maternal age was negatively associated with the presence of a febrile infection (malaria or non-malaria) in 6-month-old infants (Table 4). Given the number of tests, these results must be carefully interpreted and therefore differences with *p*<0.01 were considered significant. In this context, hypergammaglobulinemia and maternal IgG levels to MSP2-FC27 tended to be negatively and positively associated with the occurrence of an infant infection, respectively. Neither the transfer of other malaria-specific IgG nor of total IgG was associated with infection incidence. Placental malaria was not associated with 6-month-old infant infections. When infections were split into non-malaria (mainly gastrointestinal (31.6%) and respiratory (65.2%) febrile syndromes [19]) and malaria, only maternal age was negatively associated with the presence of a malaria infection in infants less than 6 months of age (Table 5).

**Table 4:** Logistic regression model showing the factors involved in infant infections.

Factors	coeff ^a^	*p*-value
Hypergammaglobulinemia	-0.457 [-0.849;-0.064]	0.023
Maternal age	-0.049 [-0.082;-0.017]	**0.003**
Maternal IgG to MSP2-FC27	0.291 [0.011;0.570]	0.042

^a^: coeff [95% confidence interval]: coefficient determined by the logistic model. A positive (negative) coefficient corresponds to a positive (negative) association between two variables; in bold: *p*<0.01.

**Table 5:** Multinomial logistic regression model showing the factors involved in infant malaria/non-malaria infections.

Infections	Factors	coeff ^a^	*p*-value
Non-malaria infection	Hypergammaglobulinemia	-0.474 [-0.910;-0.037]	0.033
	Maternal age	-0.041 [-0.076;-0.005]	0.025
	Maternal IgG to MSP2-FC27	0.314 [0.008;0.620]	0.044
Malaria infection	Hypergammaglobulinemia	-0.413 [-1.024;-0.198]	0.185
	Maternal age	-0.072 [-0.122;-0.021]	**0.005**
	Maternal IgG to MSP2-FC27	0.229 [-0.200;0.658]	0.296

^a^: coeff [95% confidence interval]: coefficient determined by the logistic model. A positive (negative) coefficient corresponds to a positive (negative) association between two variables; in bold: *p*<0.01.

## Discussion

This study aimed to describe the neonatal transfer of maternal antibodies specific to malaria blood-stage antigens. It showed that i) PM was associated with a lower CORD/CIRC ratio of malaria-specific IgG, ii) maternal IgG transfer differed depending on malaria antibody specificity, iii) cord blood antimalarial IgG levels were equivalent after adjustment for hypergammaglobulinemia and PM and iv) older maternal age was associated with fewer malaria infections in infants.

One limitation of this study may be the definition of PM based on light microscopy. As antibody transfer increases at the end of pregnancy, an active placental infection could reinforce the strong association with lower antibody transfer. Using more sensitive methods such as histopathology or molecular biology, which can determine low parasitemia in placental infections, could refine the results. However, a recent study in which placental infection was defined by light microscopy, quantitative PCR or histological examination provided results comparable to those in this study [11].

The present study confirmed that hypergammaglobulinemia or high levels of specific antibodies in maternal blood were associated with a decrease in the CORD/CIRC ratio after adjustment for PM. Regarding the mechanism of placental antibody transfer, it has been suggested that the FcRn expressed by syncytiotrophoblasts in placenta binds IgG with subsequent internalization of the FcRn-IgG complex in endosomal compartments and release of maternal IgG in fetal circulation [20,21]. The amount of IgG transmitted depends on the number of cell-surface receptors because unbound IgG are digested by lysosomal enzymes inside the vesicles, as has been suggested [22]. Some IgG may also be retained in the maternal compartment due to host differences in the functionality and/or density of the FcRn [23] regardless of malaria. We also hypothesize that the parasitized erythrocytes could adhere to syncytiotrophoblast vesicles, where the IgG binding site is assumed to be [21,24], physically impairing IgG capture from the maternal compartment. To our knowledge, no evidence exists to prove that the receptors of parasitized erythrocytes (chondroitin sulfate A) and vesicles are located at different parts of the syncytiotrophoblast. Finally, maternal IgG could be monopolized in the intervillous space to combat a local malaria infection, contributing to decreasing transplacental IgG transfer.

Several studies, including one that uses the same cohort as the current study [25–29], established that infants born to mothers with PM detected at delivery were more susceptible to malaria infections in infancy than others. This occurred independently of similar specific antibody levels measured in the cord blood of infants born to infected and noninfected mothers [14], suggesting that the PM-associated decrease of maternal IgG transfer may not explain the higher risk of malaria infection observed in these infants and may instead be a marker of maternal exposure to parasites [11].

These results show that maternal IgG to MSP2-FC27 tended to be positively related to the number of non-malaria infections in infants under 6 months old, independently of PM. Given the few tests performed, these results should be interpreted with caution. However, a positive association between the transfer of IgG1 to AMA1 as well as IgG to parasite lysate and malaria incidence during the 1^st^ year of life was also observed in another study [11]. Similarly, maternal IgG levels to variant surface antigens expressed by chondroitin sulfate A-adhering parasites were negatively correlated to time before the first parasitemia in Cameroonian infants and positively correlated to mean parasite density, suggesting a nonprotective role [30]. Lastly, a Ghanian study conducted showed that high maternal IgG levels to MSP1-19, MSP2-FC27 and Pf155/RESA were associated with a higher risk of infant infection [31].

Based on these studies and the present work, it seems that maternal IgG specific to malaria is a marker of maternal exposure to *P*. *falciparum*. Several studies have already shown that maternal antibodies can inhibit infant responses to different diseases, such as measles or tetanus [32–34]. How maternal antimalarial IgG influences malaria or non-malaria infection incidence in infants remains unclear and needs further investigation.

Interestingly, we found that the older a pregnant woman was, lower the number of malaria infections of her infant was, suggesting that effective maternal immunity acquired with age can be transmitted to offspring. This raises questions on how the immune response evolves in pregnancy and we speculate on the role of one or several maternal molecules. These molecules must i) be produced differently with age and ii) have the property of either crossing the placental barrier or inducing, by their presence at the maternal—fetal interface, tolerance/protection against malaria infections during the first 6 months of life. Cytokines and antibodies are two types of molecules with these properties, in agreement with a definition of transplacental immune regulation formulated by Santner *et al*. as “the concept that during pregnancy, significant cross-talk occurs between the maternal and fetal immune system with potential long-term effects for both the mother and child” [35]. Few clues are available in the literature, particularly IL-10, which is a cytokine with known immunomodulatory properties [36]. Newborns born to young mothers have been shown to present higher fetal plasma IL-10 levels than newborns born to older mothers [37]. Similarly, maternal IL-10 levels have been negatively associated with parity [38], which may reasonably be related to maternal age. Evidence for alignment between maternal and fetal cellular immunity has been provided, implying T regulatory cells and IL-10 concentrations [35]. Lastly, higher IL-10 levels were found in malaria-exposed, non-sensitized newborns compared to sensitized, unexposed newborns, with this phenotype persisting during childhood [28]. Based on these studies, we hypothesize that IL-10 levels in the sera of mother and her fetus may act as a molecular contributor increasing protection of the infant against malaria infection depending on maternal age.

This study did not explore the functionality of maternal antibodies, possibly one of the immune parameters evolving with maternal age (in that the total IgG quantity was not associated with maternal age resulting from a linear regression coefficient = −0.0038; *p* = 0.598). Transfer of functional malaria antibodies to the fetus has been demonstrated by measuring their ability to inhibit parasite growth in cord blood and infant blood, but the experiment did not consider maternal blood or maternal age [10]. In fact, the sole antibodies that evolve in a parity- (and therefore age-) dependent manner in malaria-endemic areas are those directed to the variant surface antigen responsible for placental adhesion of infected erythrocytes, represented by the VAR2CSA variant of the *P*. *falciparum* erythrocyte membrane protein 1 (PfEMP1) antigen [39–41]. Maternal transfer of functional antibodies or anti-VAR2CSA antibodies to the fetus and the cytokine levels in fetal plasma depending on maternal age should be further investigated to provide new clues on whether or not maternal age plays a protective role in infant health.

In conclusion, This study confirmed lower maternal transfer of IgG directed to several asexual stages of *P*. *falciparum* in placental malaria. Why the degree of maternal antimalarial IgG transfer was not related to the incidence of febrile infections in infants’ first 6 months of life, but instead maternal age, needs further investigation.
